# Prognostic Impact of Resection Margin Status on Distal Pancreatectomy for Ductal Adenocarcinoma

**DOI:** 10.3390/curroncol29090515

**Published:** 2022-09-14

**Authors:** Maia Blomhoff Holm, Caroline Sophie Verbeke

**Affiliations:** 1 Department of Pathology, Faculty of Medicine, University of Oslo, 0372 Oslo, Norway; 2 Department of Pathology, Oslo University Hospital, 0424 Oslo, Norway

**Keywords:** pancreatic cancer, margin, pathology, prognosis

## Abstract

Pancreatic cancer is associated with a poor prognosis. While surgical resection is the only treatment option with curative intent, most patients die of locoregional and/or distant recurrence. The prognostic impact of the resection margin status has received much attention. However, the evidence is almost exclusively related to pancreatoduodenectomies, while corresponding data for distal pancreatectomy specimens are limited. The key data, such as the rate of microscopic margin involvement (“R1”), the site of margin involvement, and the impact of R1 on patient outcome, are divergent between studies and do not currently allow any general conclusions. The main reasons for the variability in the published data are the small size of the study cohorts and their heterogeneity, as well as the marked divergence in pathology examination practices. The latter is a consequence of the lack of concrete guidance, both for grossing and microscopic examination. The increasing administration of neoadjuvant chemo(radio)therapy introduces a further factor of uncertainty as the conventional definition of a tumour-free margin (“R0”) based on 1 mm clearance is inadequate for these specimens. This review discusses the published data regarding the prognostic impact of margin status in distal pancreatectomy specimens along with the challenges and uncertainties that are related to the assessment of the margins.

## 1. Introduction

The prognosis of pancreatic ductal adenocarcinoma (PDAC), commonly referred to as pancreatic cancer, remains poor. Surgical resection combined with systemic therapy offers the only chance of long-term survival and a potential cure [1]. Consequently, the resection margin status—a proxy for local radicality—and its prognostic impact have increasingly received attention during the past two decades [2]. Most of the knowledge regarding margin status comes from studying resection specimens following pancreatoduodenectomy for PDAC in the pancreatic head. Based on the collective experience that has been gathered to date, three main observations can be made. First, the R1 rate—that is, the proportion of resections for PDAC with a microscopically positive resection margin—varies considerably between studies and centres, from below 20 to over 80% [3,4]. Second, as (inter-)national pathology guidelines lack instructions regarding specimen grossing and partly differ in their recommendations as to which specimen margins and surfaces are to be examined, considerable divergence in examination practice continues to exist. Third, it is only in recent years that most, but not all, pathologists have begun to use the same definition for reporting microscopic margin involvement. The longstanding lack of consensus regarding the pathology examination and reporting of the margin status explains to a large extent the considerable variation in the reported R1 rates [3]. Importantly, the incomparability of the published R1 rates has profoundly affected the analysis of the prognostic significance of the margin status on patient outcome [2,5].

Distal pancreatectomy for cancers in the pancreatic body and tail is a less commonly performed procedure compared to pancreatoduodenectomy because PDAC in the pancreatic body and tail is often more advanced at the time of diagnosis such that surgery is not a treatment option [6]. Resection margins in distal pancreatectomy specimens partly differ from those in pancreatoduodenectomy specimens, given the differences in anatomy and resected structures. In this review, various aspects of the examination and prognostic relevance of the margin status in distal pancreatectomy specimens for PDAC will be reviewed and discussed in comparison with the evidence that exists on this topic for pancreatoduodenectomy specimens.

## 2. Pathology Examination of the Margin Status—Specimen Grossing

While it is well established that the gross pathology examination is a key determinant of the accuracy of margin status reporting for pancreatoduodenectomy specimens with PDAC [3], only a single study has investigated this important quality issue for distal pancreatectomy specimens. Sahakyan et al. reported that the R1 rate increased from 60.4% to 83.1% following the implementation of a detailed pathology protocol that included a comprehensive, standardized grossing procedure for distal pancreatectomy specimens [7]. Specimen grossing consists of three steps: specimen dissection, macroscopic examination, and tissue sampling, and the latter in particular is a key determinant of the accuracy of margin assessment. Because “R1” is by definition a microscopic finding, and considering that the invasive front of PDAC is often difficult to discern on naked-eye inspection, extensive tissue sampling from the tumour onto the adjacent specimen surface(s) increases the likelihood of detecting microscopic margin involvement. Accordingly, a positive correlation between the number of such tissue samples and the R1 rate has been reported for pancreatoduodenectomy specimens [8]. In spite of this rationale, current (inter-)national guidelines do not provide any recommendations regarding the extent of tissue sampling for either pancreatoduodenectomy or distal pancreatectomy specimens.

National and international guidelines also lack clear recommendations for the grossing of distal pancreatectomy specimens. However, a detailed protocol has been published in the context of a recent clinical trial [9]. Because the anatomy of distal pancreatectomy specimens is straightforward, and given the oblong shape of the body and tail of the pancreas, most pathologists employ the same dissection technique that is based on serial sagittal slicing. Most pathologists also examine the same pancreatic surfaces, that is, the anterior and posterior surfaces as well as the pancreatic transection margin, although some variation may exist. Remarkably, according to guidelines issued by US professional bodies, investigation of the anterior and posterior surfaces is recommended, but not required, for pancreatoduodenectomy specimens [10,11,12]. It is not specified whether this also applies to distal pancreatectomy specimens or, if required for the latter, what the rationale for the different approach could be. Divergence in practice also exists regarding the transection margins of the splenic artery and vein, the examination of which is recommended only by the National Comprehensive Cancer Network (NCCN) [12], but is not mentioned in other guidelines [10,13,14,15].

In the case of *extended* surgical procedures, the grossing technique employed is likely highly variable between centres and individual pathologists. Extended, multivisceral resection may be required if PDAC in the pancreatic body and tail infiltrates neighbouring organs and structures (Table 1) [7,16,17,18,19,20,21]. While dissection of these complex specimens may be challenging, no guidance is provided by the main professional bodies. Equally lacking are recommendations regarding the assessment of the specimen margins that are created by the resection of additional organs and structures.

**Figure 1:**
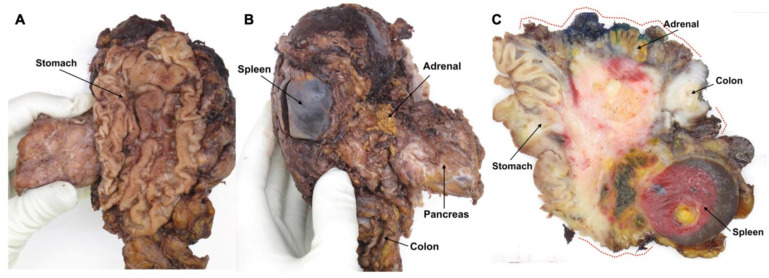
Resection margins in an extended distal pancreatectomy specimen that includes a part of the stomach (**A**) and the left adrenal gland and a segment from the left colonic flexure (**B**). Sagittal slicing reveals a large pancreatic cancer that invades all three additionally resected organs and grows close to the circumferential margin of the soft tissue between these structures (*red dotted lines*) (**C**).

Most commonly, only the transection margins of the latter, for instance, a segment of large bowel, are examined and found to be clear, while the circumferential surface of the adherent tissues between the pancreas and colon is usually not included in the examination but may well be involved (Figure 1). In order to avoid disruption of these circumferential surfaces and at the same time preserve the spatial relationship between the cancer, the pancreas, and the additionally resected structures, serial slicing of the specimen is best carried out en bloc. While photodocumentation by means of close-up photographs of the specimen slices should always be part of standardised specimen grossing, this is especially advisable in the case of extended resection specimens as it allows review of the macroscopic findings and direct correlation with histology.

## 3. Pathology Examination of the Margin Status—Microscopic Examination

### 3.1. Definition of R1

According to the TNM classification of malignant tumours issued by the Union for International Cancer Control (UICC) and the American Joint Committee on Cancer (AJCC), R1 is defined as the presence of microscopic residual disease after treatment [11,22]. Yet, in practice, R1 is used in a narrower sense, i.e., to denote microscopic residual cancer in the surgical bed. However, as the latter cannot be examined, it is not possible to establish with certainty whether cancer cells were left in situ following surgery. The R-status therefore reflects the *likelihood* of microscopic residual disease, and its assessment is based on the clearance to the margins. The exact size of this clearance, which defines “R1”, has been the subject of controversy, with the proposed minimum distance to the margin ranging from 0 mm to 1 mm or more [23,24,25]. Following a debate that has been ongoing for more than a decade, an international consensus has finally been reached, according to which a clearance of 1 mm or less is regarded as R1 [10,11,13,14,15]. This definition is a mere adoption from rectal cancer, for which a strong correlation between local recurrence and a margin clearance of 1 mm or less has been convincingly demonstrated. However, differences between the growth patterns of rectal and pancreatic cancer, with the latter growing much more dispersedly than the former, indicate that a larger clearance may be more appropriate for R1 in PDAC [26].

As a consequence of the lack of consensus that existed until recently, R1 rates published in the literature may be based on different R1 definitions, which, inevitably, has had an impact on the reported data. Indeed, several recent studies on cancers in the pancreatic head report that the R1 rate increases up to threefold if R1 is based on ≤1 mm clearance instead of 0 mm [27,28,29,30]. Similarly, for PDAC in the pancreatic body and tail, Hank and colleagues reported an R1 rate of 76.5% with invasion within 1 mm to the margin, and 53.6% with direct margin involvement (0 mm clearance) [31].

### 3.2. R1 and the Mode of Cancer Spread Close to a Margin

**Figure 2:**
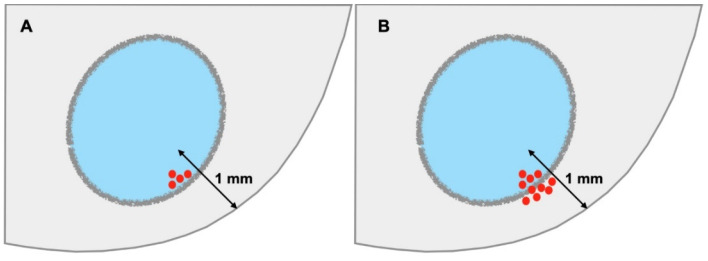
R1 and mode of cancer spread. A lymph node metastasis separated by ≤1 mm from the margin is not considered R1 as the tumour lies within a segregated microanatomical compartment (**A**). Only when there is extranodal growth, i.e., cancer cells grow outside the boundaries of the lymph node compartment at 1 mm or less from the margin, is the risk for residual cancer in the surgical bed increased, and the finding is to be reported as R1 (**B**).

Confusion amongst pathologists also prevails as to whether the mode of cancer spread is relevant to diagnosing microscopic margin involvement. While the 1 mm definition clearly pertains to cancer cells invading the tissues close to a margin, does it also apply when cancers cells are present within lymphovascular channels, perineural clefts, or lymph nodes? Following the principles of TNM reporting, R1 only pertains to direct tumour invasion, that is, cancer growth within the stroma, for the following two reasons. First, cancer spreading along lymphatic, vascular, or neural routes portends a risk for locoregional or distant tumour recurrence, which may be outside the surgical bed and is independent of surgical resection. Second, the risk for cancer recurrence associated with cancer spread along pre-existing structures is independent of the proximity of the latter to a margin. For example, lymphatic tumour permeation in the centre of the tumour is associated with the same risk for spread to regional lymph nodes as when it were to occur within 1 mm to a margin. Consequently, only when cancer cells breach the anatomical boundary of the particular compartment, e.g., when a lymph node metastasis shows extranodal tumour growth, and cancer cells start invading the surrounding tissue, does the 1 mm rule become applicable (Figure 2).

## 4. Prognostic Impact of Resection Margin Status in Distal Pancreatectomy

### 4.1. Limitations of Current Published Data

The published results regarding the prognostic impact of margin status in PDAC resection specimens are conflicting, with the main reason for the continued controversy being, as outlined above, the divergence in pathology practice [5,30]. Indeed, similar to the variation that has been observed for pancreatoduodenectomies, the R1 rates reported for distal pancreatectomies range widely, from below 10 to over 80% (Table 2). In addition to the divergence in pathology examination, further factors limit the relevance of the few studies that hitherto have been published: the small size of the study cohorts and their heterogeneity in terms of surgery (inclusion of total pancreatectomy and pancreatoduodenectomy), treatment (inclusion of neoadjuvantly treated patients), and tumour histology (inclusion of PDAC associated with intraductal papillary mucinous neoplasia). As a consequence, the evidence is currently insufficient to draw valid conclusions (Table 2).

In the studies by Sahakyan [7,33], Paye [18], Chang [23], and Shimada [35] and colleagues, the overall resection margin status was not a prognostic factor for survival in patients undergoing distal pancreatectomy for PDAC. Based on a meta-analysis combined with the analysis of a small institutional series, Demir and colleagues reported that margin status correlated with outcome in patients following pancreatoduodenectomy but not in the small subgroup of patients that had undergone distal pancreatectomy [32]. In contrast, in a large international cohort of distal pancreatectomies recently published by Korrel and colleagues, R0 resection was associated with improved overall survival [16]. Similarly, Hank and colleagues demonstrated that clearance of ≥1 mm was an independent determinant of overall survival following distal pancreatectomy [31]. Additionally, earlier studies by Yamamoto [34] and de Rooij [17] found margin status to be of prognostic significance for PDAC in the body and tail of the pancreas. However, the latter two studies included multiple resections with macroscopic margin involvement, so-called R2 resections (see Table 2), which are known to have a considerable negative influence on outcome and may have obfuscated the results.

### 4.2. Burden of Residual Disease and Time to Recurrence

When reflecting on the prognostic significance of margin status in PDAC resection specimens, two key issues are often not considered. For one, the majority of patients with PDAC already have lymph node metastasis, perineural, and/or vascular invasion at the time of surgery, which may lead to disseminated disease and death before residual disease at the resection site becomes clinically manifest. For instance, in the study by Sahakyan and colleagues, R1 was a significant prognostic factor for survival in univariable analysis, but did not reach significance as an independent predictor for survival in multivariable analysis, unlike lymph node ratio and perineural invasion [7]. Similarly, others reported that lymph node status and perineural invasion were stronger determinants of outcome in PDAC than R-status [36,37]. Indeed, it is conceivable that R-status may only be relevant for long-term outcomes in the absence of other (stronger) risk factors for locoregional or distant spread.

A further, largely ignored aspect that determines the time to local recurrence is the burden of residual disease, that is, the quantity of cancer cells that are left behind in the surgical bed. Microscopic margin involvement, be it in only a single small focus or in several foci and possibly at multiple margins, is indiscriminately reported as R1, although the residual tumour burden in the surgical bed is likely different in these scenarios. For pancreatoduodenectomy specimens, it is well documented that margin involvement is often multifocal [8,24,38,39], but this has been hardly analysed in specimens following distal pancreatectomy. At present, only two studies investigated the number of margins involved in distal pancreatectomy specimens, and they report that more than one margin was involved in 27.4 and 3.4% of the cases, respectively (based on an R1 definition of ≤ 1 mm and 0 mm clearance, respectively) [7,23]. If, and in how far, multifocal margin involvement and the quantity of microscopic residual tumour in the surgical bed affect outcomes is currently unknown. A further obstacle to the study of the association between residual tumour burden and patient outcome is the lack of a clear distinction between microscopic and macroscopic residual disease, that is, R1 and R2. By common agreement, the latter is diagnosed at the discretion of the surgeon, while pathologists do not usually report R2, even if they observe tumour growth within 1 mm to a margin not only in a discrete focus but in a more extensive area. In the future, with the advent of artificial intelligence-assisted cancer reporting, semiquantitative assessment of the tumour burden at the margins may possibly provide clinically more relevant information than the current binary reporting of R1 or R2.

### 4.3. Prognostic Impact of R1 at Specific Margins

For pancreatoduodenectomy specimens, it has been suggested that tumour involvement of some resection margins, in particular of the margin facing the superior mesenteric artery, has a stronger negative impact on patient outcome than that of other margins [28,38,40,41]. A possible explanation for this claim could be that margin involvement is a proxy for tumour invasion of a microanatomic compartment that is particularly rich in blood vessels, lymphatic channels, or nerves, which is the case for the peripancreatic fat facing the superior mesenteric artery [42]. As such, the impact on survival may well be due to the increased risk of dissemination along these structures rather than the consequence of cancer cells being present close to the resection margin. In distal pancreatectomy specimens, a high density of lymphatic channels and nerves may be found around the splenic artery and vein. While there has been much focus on the improvement of surgical techniques in order to reduce the R1 rate at the margins facing the superior mesenteric artery and vein during pancreatoduodenectomy, tumour growth around the splenic artery and vein and the adjacent posterior margin of distal pancreatectomy specimens has thus far received less attention. The only data that are currently published demonstrate that tumour involvement of the transection margin of the splenic artery and vein occurs in 5.6% of the cases, while the posterior margin is positive in 55.6%. Of note, neither correlated with prognosis [7].

The transection margin facing the pancreatic remnant has traditionally received much attention, both for pancreatoduodenectomy and distal pancreatectomy procedures. As opposed to the other margins, it is a widespread practice to examine the pancreatic transection margin intraoperatively by frozen section, either routinely or dependent on the surgeon´s evaluation of the transection margin and the proximity of the tumour to the margin. Two studies report that involvement of the pancreatic transection margin occurred in 12.9 and 25.7% of distal pancreatectomies, respectively [7,23]. These highly divergent observations are difficult to explain, especially when considering that the study with the lowest R1 rate used ≤1 mm clearance to report R1, whereas in the study with the significantly higher R1 rate, microscopic margin involvement was defined by 0 mm clearance. Of note, in neither of the studies was involvement of the transection margin of the pancreas found to have an impact on patient outcome.

### 4.4. Involvement of the Anterior Surface

**Figure 3:**
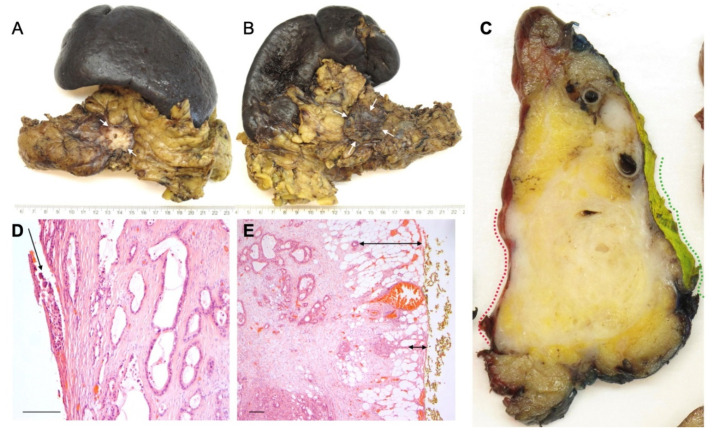
Involvement of the anterior pancreatic surface and Gerota’s fascia by this large tumour in the pancreatic body is suspected macroscopically, both on external inspection ((**A**) anterior surface, *arrows*, and (**B**) posterior surface with Gerota’s fascia, *arrows*) and on a sagittal specimen slice ((**C**) anterior surface and Gerota’s fascia, *red and green dotted lines*, respectively). Histology confirms tumour breaching of the anterior surface ((**D***) arrow*) and infiltration within 1 mm of the posterior surface of Gerota’s fascia ((**E**) *arrows*). Magnification: 400× (**D**), 200× (**E**). Scale bar (**D**,**E**): 200 microns.

It has been suggested that the anterior pancreatic surface in pancreatoduodenectomy specimens is also of prognostic importance [43,44]. Notably, the anterior pancreatic surface does not represent a true resection margin but is a peritoneum-lined anatomical surface. Therefore, involvement of this surface (R1) requires that the peritoneal lining is breached by the cancer cells (i.e., 0 mm clearance) (Figure 3). Hence, tumour involvement of the anterior pancreatic surface represents a different biological process, also because breaching of the peritoneal lining gives the tumour cells access to the lesser sac and the possibility of spreading in the peritoneal cavity. In contrast to PDAC, breaching of the peritoneal lining in colorectal cancer is reflected by T-stage, not R-stage [22]. In spite of the differences in cancer biology and clinical outcome, involvement of the anterior pancreatic surface is only rarely recorded separately, and usually included in the overall circumferential resection margin status in pathology reports on PDAC specimens. Of the currently published studies that assessed the prognostic impact of margin status following distal pancreatectomy (Table 2), only a single study reported separately on the anterior surface [7]. Interestingly, in this study, the anterior surface was found to be prognostically relevant, while this was not the case for the other margins that were examined (posterior margin, vascular transection margin, and pancreatic transection margin). Therefore, it cannot be excluded that the prognostic impact of the overall resection margin status, described by the abovementioned studies [16,17,31,34], was in fact determined by the involvement of the anterior surface of the pancreas.

### 4.5. Prognostic Impact of Resection Margin Status on Extended Distal Pancreatectomy

Because PDAC in the pancreatic body and tail is more often advanced at the time of diagnosis than cancer in the head of the pancreas, contiguous organs may be involved and require an extended distal pancreatectomy with en bloc resection of the affected structures in order to obtain macroscopically negative margins. These extended surgical procedures account for up to 30% of all distal pancreatectomy specimens [7,17,18,19,20,21,45,46]. As outlined above, pathology examination of these complex surgical specimens may be challenging, and concrete guidance as to how to undertake the examination is currently lacking. Yet, the impact of the accuracy of the pathology examination was demonstrated by a recent study in which the implementation of a meticulous, standardized examination protocol increased the R1 rate for extended resection specimens from 62.5 to 100% [7]. While the study series was relatively small (117 distal pancreatectomies, of which 34 were extended procedures), it showed that the R1 rate, both overall and specific for the anterior surface and posterior margin (but not the transection margin), was higher following extended than standard surgical procedures. The proportion of cases with microscopic involvement of more than one margin was also significantly higher. Patient outcome was found to be worse following extended distal pancreatectomy, but given that the meticulous, fully standardized pathology approach detected microscopic margin involvement in all the extended specimens, the impact of R1 on outcome remains uncertain. Currently, only three studies include a large number of extended distal pancreatectomies for PDAC [16,45,46], and while two of these also report on a worse outcome for this group, specific data on the margin status are lacking [16,46]. A few smaller studies (*n* < 50) also observed a dismal prognosis following extended surgery, but they suffer from non-standardized pathology examination and lack R1 rates for the extended procedures [18,21,47].

While resection of Gerota’s fascia is currently not part of a standard distal pancreatectomy, a recent study revealed that 30% of surgeons include this step routinely with the aim of obtaining an R0 resection at the posterior margin [16]. Surprisingly, the R1 rate for the circumferential margins was found to be higher in cases with Gerota’s fascia resection (78.1 versus 54.4% for standard distal pancreatectomy). However, due to the combined reporting on the margin status in this study, it was not possible to assess to what extent the higher R1 rate was due to involvement of the anterior surface, the posterior margin, or both. Indeed, bearing in mind the fairly small size of the pancreatic body and tail in the anteroposterior plane, and the fact that PDAC typically measures 3 cm or more at the time of diagnosis [7,16,45,48], tumours quite commonly occupy the entire width of the pancreas, threatening both the anterior surface and posterior margin (Figure 3). Interestingly, Gerota’s fascia resection was independently associated with improved overall survival, which may point at the clinical benefit of including this additional step in the surgical procedure [16].

## 5. Resection Margin Status and Neoadjuvant Treatment

**Figure 4:**
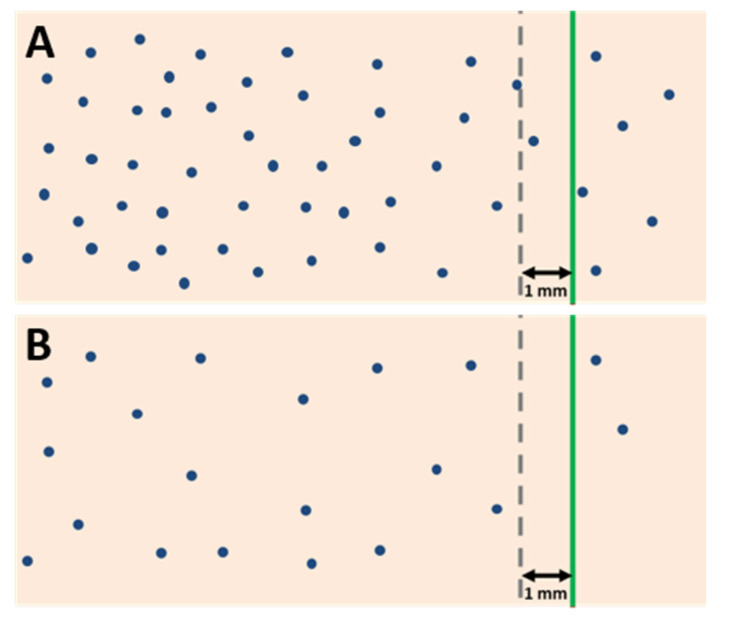
Margin clearance in treatment-naïve PDAC (**A**) and in PDAC following neoadjuvant therapy (**B**). A treatment-naïve tumour with clearance ≤ 1 mm is considered R1. However, following neoadjuvant therapy, a clearance of >1 mm does not preclude the presence of residual cancer in the surgical bed (R1) because, due to partial tumour regression, the distance between residual cancer cells may become considerably larger (*blue dots:* cancer cells, *green line:* resection margin, *grey interrupted line:* 1 mm clearance).

In recent years, there has been a considerable increase in the use of neoadjuvant chemo- and/or radiotherapy for borderline resectable, locally advanced, and primary resectable PDAC. Consequently, the impact of neoadjuvant therapy on the assessment and prognostic significance of resection margin status has become a highly relevant, yet little discussed, issue [49]. Reporting of the margin status following neoadjuvant therapy is not without its challenges. First, areas with viable residual cancer and those with tumour regression are often macroscopically indistinguishable. It is therefore crucial that tissues at the resection margins are completely embedded unless they are entirely normal-appearing and well clear of the tumour bed. Furthermore, as mentioned above, margin status assessment is based on the measurement of the minimum distance between the tumour cells and the specimen margins in order to evaluate the likelihood of microscopic residual disease in the surgical bed. However, it is important to note that the minimum clearance required to distinguish between microscopic margin involvement and a free margin is dependent on the growth pattern of the tumour: the more dispersedly a cancer grows, the larger the clearance required for the risk of residual microscopic disease to be negligible and for the margin to be considered clear (R0). Since neoadjuvant therapy leads to a loss of cancer cells, the distance between the remaining viable cancer cells may increase in areas with tumour regression, and so may the clearance to the margins. As a direct consequence, the proportion of cases with a clearance >1 mm is often high [50,51,52]. Yet, even when the clearance to the margins exceeds 1 mm, viable cancer cells may be present in the surgical bed because treatment effect is often patchy (Figure 4). Consequently, a minimum clearance of >1 mm, as currently recommended by (inter-)national guidelines to report “R0”, does not reliably reflect the absence of residual microscopic disease in the surgical bed in patients who underwent neoadjuvant treatment.

This important issue needs to be considered when interpreting study results regarding the prognostic impact of resection margin status. The fact that several of the studies on resection margins in distal pancreatectomy included both treatment-naïve and neoadjuvantly treated patients in varying proportions (Table 2) may have contributed to the diverse and partially conflicting results. Despite the increasing use of neoadjuvant treatment for PDAC, preoperative administration of chemo(radio)therapy for tumours in the body and tail of the pancreas is markedly lower than for those in the pancreatic head [53]. Hence, there is currently only one large study that investigated the clinical impact of resection margin status exclusively in PDAC patients who underwent neoadjuvant treatment followed by distal pancreatectomy [54]. The results indicate that R0 may confer a survival benefit, also following neoadjuvant therapy. However, the study suffers from a number of limitations, including the use of less effective chemotherapy regimens, such that further studies are needed for more conclusive evidence. Otherwise, the literature on neoadjuvant therapy combined with distal pancreatectomy consists of a few small studies that focus mainly on borderline resectable or locally advanced PDAC with involvement of major arterial vessels [55,56,57]. Overall, in view of the paucity of studies, the heterogeneity and small size of the study cohorts, and the divergence in pathology practice, results are sparse and conflicting, and, thus far, they do not present a coherent picture.

## 6. Conclusions

While the prognostic impact of resection margin status in PDAC is much debated, most of our current insight is largely limited to cancer in the pancreatic head resected by pancreatoduodenectomy. To date, studies on margin status following distal pancreatectomy are few in number and often limited by small, heterogeneous study cohorts and non-standardized pathology. Hence, data on the prognostic impact of resection margin status in distal pancreatectomy specimens are scarce and partially conflicting. Moreover, there is a lack of information on more specific issues, leaving clinically relevant questions unanswered regarding the prognostic impact of individual margins and the extent of residual disease and margin involvement following neoadjuvant treatment as well as extended surgical procedures. As interest in the margin status following distal pancreatectomy is increasing, there is a need for dedicated larger studies with uniform patient cohorts regarding treatment status and with fully standardized pathology examination. In order to achieve this, the publication of international pathology examination protocols with detailed recommendations for meticulous grossing and microscopic examination of distal pancreatectomy specimens is required to ensure the comparability of the data. Experience from ongoing multicentre trials on PDAC in the pancreatic body and tail may bring to the fore the need for standardization and trigger efforts to reach an international consensus [58].

## Figures and Tables

**Table 1:** Additional structures that may be resected during extended distal pancreatectomy procedures.

Gerota’s fascia
Adrenal gland
Stomach
Small bowel
Large bowel
Kidney
Large blood vessels

**Table 2:** Studies that investigated the prognostic impact of resection margin status in distal pancreatectomy specimens. Only studies based on a cohort size of more than *n* = 40 and a study period later than 2006 are included.

Study	n	R1 Rate (%)	R1 Definition	Dissection Technique	NAT (%)	Prognostic Impact of R-Status
**Sahakyan 2022** [7] Entire cohort Period 1						
	124	73.4	≤1 mm		7.3	No ^1^
	53	60.4		Sagittal slicing, not specified		
Period 2	71	83.1		Sagittal slicing, standardized		
**Korrel 2021 [16]**	1200	43.2	<1 mm	Sagittal slicing, standardized	10.7	Yes
**Hank 2018 [31]**	218	71.1	<1 mm	Sagittal slicing, standardized	-	Yes
**Demir 2018 [32]** (Institutional cohort)	44	36.4	0 mm	Not specified	-	No
		59.1	<1 mm			
**Sahakyan 2017 [33]**	186	9.5	0 mm	Not specified	1.0	No
**De Rooij 2016 [17]**	141	45.0	<1 mm	Not specified	-	Yes
		(5.0 R2)				
**Paye 2015 [18]** (Multicentre study)	278	25.2 ^2^	<1 mm	Not specified	7.2	No
**Yamamoto 2010 [34]**	73 ^3^	16.4	Not specified	Not specified	-	Yes
		(8.2 R2)				
**Chang 2009 [23]**	70	47.1	0 mm	Not specified	-	No
		57.2	<1 mm			
**Shimada 2006 [35]**	88^4^	25.0	Not specified	Not specified	-	No

^1^ Prognostic impact observed only for involvement of the anterior surface, not for other margins nor for the overall margin status. ^2^ Also includes R2 resections. ^3^ Including Appleby’s (*n* = 3), total pancreatectomy (*n* = 2), and pancreatoduodenectomy (*n* = 2) procedures. ^4^ Including Appleby’s procedure (*n* = 12). For carcinomas of the body and tail, Gerota’s fascia and the left adrenal gland were removed even in the absence of apparent invasion. NAT, neoadjuvant therapy.

## References

[1] Strobel O., Neoptolemos J., Jäger D., Büchler M.W. (2019). Optimizing the outcomes of pancreatic cancer surgery. Nat. Rev. Clin. Oncol..

[2] Niesen W., Hank T., Büchler M., Strobel O. (2019). Local radicality and survival outcome of pancreatic cancer surgery. Ann. Gastroenterol. Surg..

[3] Chandrasegaram M.D., Goldstein D., Simes J., Gebski V., Kench J.G., Gill A.J., Samra J.S., Merrett N.D., Richardson A.J., Barbour A.P. (2015). Meta-analysis of radical resection rates and margin assessment in pancreatic cancer. Br. J. Surg..

[4] Verbeke C.S., Menon K.V. (2009). Redefining resection margin status in pancreatic cancer. HPB Off. J. Int. Hepato Pancreato Biliary Assoc..

[5] Leonhardt C.S., Niesen W., Kalkum E., Klotz R., Hank T., Büchler M.W., Strobel O., Probst P. (2022). Prognostic relevance of the revised R status definition in pancreatic cancer: Meta-analysis. BJS Open.

[6] Tomasello G., Ghidini M., Costanzo A., Ghidini A., Russo A., Barni S., Passalacqua R., Petrelli F. (2019). Outcome of head compared to body and tail pancreatic cancer: A systematic review and meta-analysis of 93 studies. J. Gastrointest. Oncol..

[7] Sahakyan M.A., Verbeke C.S., Tholfsen T., Ignjatovic D., Kleive D., Buanes T., Lassen K., Røsok B.I., Labori K.J., Edwin B. (2022). Prognostic Impact of Resection Margin Status in Distal Pancreatectomy for Ductal Adenocarcinoma. Ann. Surg. Oncol..

[8] Verbeke C.S., Leitch D., Menon K.V., McMahon M.J., Guillou P.J., Anthoney A. (2006). Redefining the R1 resection in pancreatic cancer. Br. J. Surg..

[9] Lof S., Rajak R., Vissers F., Korrel M., Bateman A., Verheij J., Verbeke C., Cataldo I., Besselink M.G., Abu Hilal M. (2020). DIPLOMA Approach for Standardized Pathology Assessment of Distal Pancreatectomy Specimens. J. Vis. Exp..

[10] College of American Pathologists (CAP) (2021). Protocol for the Examination of Specimens from Patients with Carcinoma of the Pancreas. https://documents.cap.org/protocols/Panc.Exo_4.2.0.2.REL_CAPCP.pdf.

[11] Kakar S., Pawlik T.M., Allen P.J., Vauthey J.N., Amin M.B., Edge S.B., Greene F.L., Byrd D.R., Brookland R.K., Washington M.K., Gershenwald J.E., Compton C.C., Hess K.R., Sullivan D.C. (2017). AJCC Cancer Staging Manual.

[12] NCCN Guidelines version 2.2021. Pancreatic Adenocarcinoma. https://www.nccn.org/professionals/physician_gls/pdf/pancreatic_blocks.pdf.

[13] The Royal College of Pathologists of Australasia (RCPA) (2020). Cancer of the Exocrine Pancreas, Ampulla of Vater and Distal Common Bile Duct. Structured Reporting Protocol (2nd ed.). https://www.rcpa.edu.au/Library/Practising-Pathology/Structured-Pathology-Reporting-of-Cancer/Cancer-Protocols/Gastrointestinal/Protocol-pancreatic-cancer.aspx.

[14] The Royal College of Pathologists (2019). Dataset for the Histopathological Reporting of Carcinoma of the Pancreas, Ampulla of Vater and Common Bileduct. https://www.rcpath.org/uploads/assets/34910231-c106-4629-a2de9e9ae6f87ac1/G091-Dataset-for-histopathological-reporting-of-carcinomas-of-the-pancreas-ampulla-of-Vater-and-common-bile-duct.pdf.

[15] Verbeke C.B., Brosens L., Campbell F., Del Chiaro M., Esposito I., Feakins R.M., Fukushima N., Gill A., Kakar S., Kench J. (2020). Carinoma of the Exocrine Pancreas Histopathology Reporting Guide.

[16] Korrel M., Lof S., van Hilst J., Alseidi A., Boggi U., Busch O.R., van Dieren S., Edwin B., Fuks D., Hackert T. (2021). Predictors for Survival in an International Cohort of Patients Undergoing Distal Pancreatectomy for Pancreatic Ductal Adenocarcinoma. Ann. Surg. Oncol..

[17] de Rooij T., Tol J.A., van Eijck C.H., Boerma D., Bonsing B.A., Bosscha K., van Dam R.M., Dijkgraaf M.G., Gerhards M.F., van Goor H. (2016). Outcomes of Distal Pancreatectomy for Pancreatic Ductal Adenocarcinoma in the Netherlands: A Nationwide Retrospective Analysis. Ann. Surg. Oncol..

[18] Paye F., Micelli Lupinacci R., Bachellier P., Boher J.M., Delpero J.R. (2015). Distal pancreatectomy for pancreatic carcinoma in the era of multimodal treatment. Br. J. Surg..

[19] Panzeri F., Marchegiani G., Malleo G., Malpaga A., Maggino L., Marchese T., Salvia R., Bassi C., Butturini G. (2017). Distal pancreatectomy associated with multivisceral resection: Results from a single centre experience. Langenbeck’s Arch. Surg..

[20] Roch A.M., Singh H., Turner A.P., Ceppa E.P., House M.G., Zyromski N.J., Nakeeb A., Schmidt C.M. (2015). Extended distal pancreatectomy for pancreatic adenocarcinoma with splenic vein thrombosis and/or adjacent organ invasion. Am. J. Surg..

[21] Sahakyan M.A., Kleive D., Kazaryan A.M., Aghayan D.L., Ignjatovic D., Labori K.J., Røsok B.I., Edwin B. (2018). Extended laparoscopic distal pancreatectomy for adenocarcinoma in the body and tail of the pancreas: A single-center experience. Langenbeck’s Arch. Surg..

[22] Brierly J.D., Gospodarowicz M.K., Wittekind D. (2017). UICC: TNM Classification of Malignant Tumors.

[23] Chang D.K., Johns A.L., Merrett N.D., Gill A.J., Colvin E.K., Scarlett C.J., Nguyen N.Q., Leong R.W., Cosman P.H., Kelly M.I. (2009). Margin clearance and outcome in resected pancreatic cancer. J. Clin. Oncol.Off. J. Am. Soc. Clin. Oncol..

[24] Jamieson N.B., Chan N.I., Foulis A.K., Dickson E.J., McKay C.J., Carter C.R. (2013). The prognostic influence of resection margin clearance following pancreaticoduodenectomy for pancreatic ductal adenocarcinoma. J. Gastrointest. Surg. Off. J. Soc. Surg. Aliment. Tract.

[25] Osipov A., Nissen N., Rutgers J., Dhall D., Naziri J., Chopra S., Li Q., Hendifar A.E., Tuli R. (2017). Redefining the Positive Margin in Pancreatic Cancer: Impact on Patterns of Failure, Long-Term Survival and Adjuvant Therapy. Ann. Surg. Oncol..

[26] Verbeke C.S., Knapp J., Gladhaug I.P. (2011). Tumour growth is more dispersed in pancreatic head cancers than in rectal cancer: Implications for resection margin assessment. Histopathology.

[27] Weyhe D., Obonyo D., Uslar V.N., Stricker I., Tannapfel A. (2021). Predictive factors for long-term survival after surgery for pancreatic ductal adenocarcinoma: Making a case for standardized reporting of the resection margin using certified cancer center data. PLoS ONE.

[28] Sohn H.J., Kim H., Kim S.J., Lee K.B., Han Y., Lee J.M., Kang J.S., Kwon W., Chie E.K., Kim H. (2022). Oncologic outcomes according to the location and status of resection margin in pancreas head cancer: Role of radiation therapy in R1 resection. Ann. Surg. Treat. Res..

[29] Crippa S., Giannone F., Schiavo Lena M., Belfiori G., Partelli S., Tamburrino D., Delpini R., Pagnanelli M., Pecorelli N., Balzano G. (2021). R Status is a Relevant Prognostic Factor for Recurrence and Survival After Pancreatic Head Resection for Ductal Adenocarcinoma. Ann. Surg. Oncol..

[30] Strobel O., Hank T., Hinz U., Bergmann F., Schneider L., Springfeld C., Jäger D., Schirmacher P., Hackert T., Büchler M.W. (2017). Pancreatic Cancer Surgery: The New R-status Counts. Ann. Surg..

[31] Hank T., Hinz U., Tarantino I., Kaiser J., Niesen W., Bergmann F., Hackert T., Büchler M.W., Strobel O. (2018). Validation of at least 1 mm as cut-off for resection margins for pancreatic adenocarcinoma of the body and tail. Br. J. Surg..

[32] Demir I.E., Jäger C., Schlitter A.M., Konukiewitz B., Stecher L., Schorn S., Tieftrunk E., Scheufele F., Calavrezos L., Schirren R. (2018). R0 Versus R1 Resection Matters after Pancreaticoduodenectomy, and Less after Distal or Total Pancreatectomy for Pancreatic Cancer. Ann. Surg..

[33] Sahakyan M.A., Kim S.C., Kleive D., Kazaryan A.M., Song K.B., Ignjatovic D., Buanes T., Røsok B.I., Labori K.J., Edwin B. (2017). Laparoscopic distal pancreatectomy for pancreatic ductal adenocarcinoma: Long-term oncologic outcomes after standard resection. Surgery.

[34] Yamamoto J., Saiura A., Koga R., Seki M., Katori M., Kato Y., Sakamoto Y., Kokudo N., Yamaguchi T. (2010). Improved survival of left-sided pancreas cancer after surgery. Jpn. J. Clin. Oncol..

[35] Shimada K., Sakamoto Y., Sano T., Kosuge T. (2006). Prognostic factors after distal pancreatectomy with extended lymphadenectomy for invasive pancreatic adenocarcinoma of the body and tail. Surgery.

[36] Tummers W.S., Groen J.V., Sibinga Mulder B.G., Farina-Sarasqueta A., Morreau J., Putter H., van de Velde C.J., Vahrmeijer A.L., Bonsing B.A., Mieog J.S. (2019). Impact of resection margin status on recurrence and survival in pancreatic cancer surgery. Br. J. Surg..

[37] Felsenstein M., Lindhammer F., Feist M., Hillebrandt K.H., Timmermann L., Benzing C., Globke B., Zocholl D., Hu M., Fehrenbach U. (2022). Perineural Invasion in Pancreatic Ductal Adenocarcinoma (PDAC): A Saboteur of Curative Intended Therapies?. J. Clin. Med..

[38] Ghaneh P., Kleeff J., Halloran C.M., Raraty M., Jackson R., Melling J., Jones O., Palmer D.H., Cox T.F., Smith C.J. (2019). The Impact of Positive Resection Margins on Survival and Recurrence Following Resection and Adjuvant Chemotherapy for Pancreatic Ductal Adenocarcinoma. Ann. Surg..

[39] Kleive D., Labori K.J., Line P.D., Gladhaug I.P., Verbeke C.S. (2020). Pancreatoduodenectomy with venous resection for ductal adenocarcinoma rarely achieves complete (R0) resection. HPB Off. J. Int. Hepato Pancreato Biliary Assoc..

[40] Zhang Y., Frampton A.E., Cohen P., Kyriakides C., Bong J.J., Habib N.A., Spalding D.R., Ahmad R., Jiao L.R. (2012). Tumor infiltration in the medial resection margin predicts survival after pancreaticoduodenectomy for pancreatic ductal adenocarcinoma. J. Gastrointest. Surg. Off. J. Soc. Surg. Aliment. Tract.

[41] Kalisvaart M., Broadhurst D., Marcon F., Pande R., Schlegel A., Sutcliffe R., Marudanayagam R., Mirza D., Chatzizacharias N., Abradelo M. (2020). Recurrence patterns of pancreatic cancer after pancreatoduodenectomy: Systematic review and a single-centre retrospective study. HPB Off. J. Int. Hepato Pancreato Biliary Assoc..

[42] Reinehr M.D., Vuille-Dit-Bille R.N., Soll C., Mittal A., Samra J.S., Staerkle R.F. (2022). Anatomy of the neural fibers at the superior mesenteric artery-a cadaver study. Langenbeck’s Arch. Surg..

[43] Nagakawa T., Sanada H., Inagaki M., Sugama J., Ueno K., Konishi I., Ohta T., Kayahara M., Kitagawa H. (2004). Long-term survivors after resection of carcinoma of the head of the pancreas: Significance of histologically curative resection. J Hepatobiliary Pancreat Surg.

[44] Tsuchiya R., Noda T., Harada N., Miyamoto T., Tomioka T., Yamamoto K., Yamaguchi T., Izawa K., Tsunoda T., Yoshino R. (1986). Collective review of small carcinomas of the pancreas. Ann. Surg..

[45] van Hilst J., de Rooij T., Klompmaker S., Rawashdeh M., Aleotti F., Al-Sarireh B., Alseidi A., Ateeb Z., Balzano G., Berrevoet F. (2019). Minimally Invasive versus Open Distal Pancreatectomy for Ductal Adenocarcinoma (DIPLOMA): A Pan-European Propensity Score Matched Study. Ann. Surg..

[46] Sulpice L., Farges O., Goutte N., Bendersky N., Dokmak S., Sauvanet A., Delpero J.R. (2015). Laparoscopic Distal Pancreatectomy for Pancreatic Ductal Adenocarcinoma: Time for a Randomized Controlled Trial? Results of an All-inclusive National Observational Study. Ann. Surg..

[47] Shin S.H., Kim S.C., Song K.B., Hwang D.W., Lee J.H., Park K.M., Lee Y.J. (2016). Appraisal of Laparoscopic Distal Pancreatectomy for Left-Sided Pancreatic Cancer: A Large Volume Cohort Study of 152 Consecutive Patients. PLoS ONE.

[48] Tran M.L., Holm M.B., Verbeke C.S. (2022). Tumour Size and T-Stage in Pancreatic Cancer Resection Specimens Depend on the Pathology Examination Approach. Cancers.

[49] Soer E.C., Verbeke C.S. (2021). Pathology reporting of margin status in locally advanced pancreatic cancer: Challenges and uncertainties. J. Gastrointest. Oncol..

[50] Schmocker R.K., Delitto D., Wright M.J., Ding D., Cameron J.L., Lafaro K.J., Burns W.R., Wolfgang C.L., Burkhart R.A., He J. (2021). Impact of Margin Status on Survival in Patients with Pancreatic Ductal Adenocarcinoma Receiving Neoadjuvant Chemotherapy. J. Am. Coll. Surg..

[51] Addeo P., Cusumano C., Dufour P., Avérous G., Bachellier P. (2022). Upfront versus resection after neoadjuvant chemotherapy for pancreatic adenocarcinomas with venous contact: Comparative analysis of operative and survival outcomes. Surgery.

[52] Villano A.M., O’Halloran E., Goel N., Ruth K., Barrak D., Lefton M., Reddy S.S. (2022). Total neoadjuvant therapy is associated with improved overall survival and pathologic response in pancreatic adenocarcinoma. J. Surg. Oncol..

[53] Nelson D.W., Chang S.C., Grunkemeier G., Dehal A.N., Lee D.Y., Fischer T.D., DiFronzo L.A., O’Connor V.V. (2018). Resectable Distal Pancreas Cancer: Time to Reconsider the Role of Upfront Surgery. Ann. Surg. Oncol..

[54] Nassour I., Adam M.A., Kowalsky S., Al Masri S., Bahary N., Singhi A.D., Lee K., Zureikat A., Paniccia A. (2021). Neoadjuvant therapy versus upfront surgery for early-stage left-sided pancreatic adenocarcinoma: A propensity-matched analysis from a national cohort of distal pancreatectomies. J. Surg. Oncol..

[55] Yoshitomi H., Sakai N., Kagawa S., Takano S., Ueda A., Kato A., Furukawa K., Takayashiki T., Kuboki S., Miyzaki M. (2019). Feasibility and safety of distal pancreatectomy with en bloc celiac axis resection (DP-CAR) combined with neoadjuvant therapy for borderline resectable and unresectable pancreatic body/tail cancer. Langenbeck’s Arch. Surg..

[56] Baumgartner J.M., Krasinskas A., Daouadi M., Zureikat A., Marsh W., Lee K., Bartlett D., Moser A.J., Zeh H.J. (2012). Distal pancreatectomy with en bloc celiac axis resection for locally advanced pancreatic adenocarcinoma following neoadjuvant therapy. J. Gastrointest. Surg. Off. J. Soc. Surg. Aliment. Tract.

[57] Yoshiya S., Fukuzawa K., Inokuchi S., Kosai-Fujimoto Y., Sanefuji K., Iwaki K., Motohiro A., Itoh S., Harada N., Ikegami T. (2020). Efficacy of Neoadjuvant Chemotherapy in Distal Pancreatectomy with En Bloc Celiac Axis Resection (DP-CAR) for Locally Advanced Pancreatic Cancer. J. Gastrointest. Surg. Off. J. Soc. Surg. Aliment. Tract.

[58] van Hilst J., Korrel M., Lof S., de Rooij T., Vissers F., Al-Sarireh B., Alseidi A., Bateman A.C., Björnsson B., Boggi U. (2021). Minimally invasive versus open distal pancreatectomy for pancreatic ductal adenocarcinoma (DIPLOMA): Study protocol for a randomized controlled trial. Trials.

